# mtDNA content in cumulus cells does not predict development to blastocyst or implantation

**DOI:** 10.1093/hropen/hoac029

**Published:** 2022-07-06

**Authors:** Álvaro Martínez-Moro, Ismael Lamas-Toranzo, Leopoldo González-Brusi, Alba Pérez-Gómez, Ester Padilla-Ruiz, Javier García-Blanco, Pablo Bermejo-Álvarez

**Affiliations:** Animal Reproduction Department, INIA, CSIC, Madrid, Spain; IVF Spain, Madrid, Spain; Animal Reproduction Department, INIA, CSIC, Madrid, Spain; Animal Reproduction Department, INIA, CSIC, Madrid, Spain; Animal Reproduction Department, INIA, CSIC, Madrid, Spain; IVF Spain, Madrid, Spain; IVF Spain, Madrid, Spain; Animal Reproduction Department, INIA, CSIC, Madrid, Spain

**Keywords:** mitochondria, mtDNA, oocyte quality, cumulus cells, granulosa cells, embryo development, developmental competence, developmental proxy, implantation

## Abstract

**STUDY QUESTION:**

Is relative mitochondrial DNA (mtDNA) content in cumulus cells (CCs) related to embryo developmental competence in humans and/or the bovine model?

**SUMMARY ANSWER:**

mtDNA content in CCs provides a poor predictive value of oocyte developmental potential, both *in vitro* and following embryo transfer.

**WHAT IS KNOWN ALREADY:**

CCs are closely connected to the oocyte through transzonal projections, serving essential metabolic functions during folliculogenesis. These oocyte-supporting cells are removed and discarded prior to ICSI, thereby providing interesting biological material on which to perform molecular analyses designed to identify markers that predict oocyte developmental competence. Previous studies have positively associated oocyte mtDNA content with developmental potential in animal models and women. However, it remains debatable whether mtDNA content in CCs could be used as a proxy to infer oocyte developmental potential

**STUDY DESIGN, SIZE, DURATION:**

mtDNA content was analyzed in CCs obtained from 109 human oocytes unable to develop to blastocyst, able to develop to blastocyst but failing to establish pregnancy or able to develop to blastocyst and to establish pregnancy. mtDNA analysis was also performed on bovine cumulus samples collected from 120 oocytes unable to cleave, oocytes developing into cleaved embryos but arresting development prior to the blastocyst stage or oocytes developing to blastocysts.

**PARTICIPANTS/MATERIALS, SETTING, METHODS:**

Human CCs samples were obtained from women undergoing IVF. Only unfrozen oocytes and embryos not submitted to preimplantation genetic testing were included in the analysis. Bovine samples were obtained from slaughtered cattle and individually matured, fertilized and cultured *in vitro*. Relative mtDNA was assessed by quantitative PCR analysis.

**MAIN RESULTS AND THE ROLE OF CHANCE:**

mtDNA content in human and bovine CCs did not differ according to the developmental potential of their enclosed oocyte. Moreover, mtDNA content in bovine oocytes did not correlate with that of their corresponding CCs.

**LARGE SCALE DATA:**

N/A.

**LIMITATIONS, REASONS FOR CAUTION:**

The lack of correlation found between mtDNA content in human CCs and oocytes was also assessed in bovine samples. Although bovine folliculogenesis, mono-ovulatory ovulation and early embryo development exhibit considerable similarities with that of humans, they may not be fully comparable.

**WIDER IMPLICATIONS OF THE FINDINGS:**

The use of molecular markers for oocyte developmental potential in CCs could be used to enhance success rates following single embryo transfer. However, our data indicate that mtDNA in CCs is not a good proxy for oocyte quality.

**STUDY FUNDING/COMPETING INTEREST(S):**

This research was supported by the Industrial Doctorate Project IND2017/BIO-7748 funded by the Madrid Region Government. The authors declare no competing interests.

WHAT DOES THIS MEAN FOR PATIENTS? Embryo viability (i.e. development and survival) plays a major role in successful reproduction and therefore improving the methods used for embryo selection is crucial to improve pregnancy rates following artificial reproductive techniques (e.g. IVF). Cumulus cells (CCs) completely surround an oocyte (egg) and provide the oocyte with essential nourishment but they are often removed prior to fertilization. Being a ‘by product’ of the IVF process, CCs can be used in analyses aimed to infer the odds for embryo survival following transfer to the uterus. In this study, we have tested whether the amount of mitochondrial DNA (mtDNA) in CCs differs between eggs that are unable to develop outside the body (*in vitro*), are able to develop *in vitro* but fail to establish pregnancy or those that are able to establish a pregnancy. Our results showed no differences in mtDNA content between the three groups, suggesting that mtDNA in CCs is of poor predictive value for the developmental potential of an egg.

## Introduction

Embryo viability plays a major role in reproductive success and, thus, the implementation of methods for embryo selection is crucial to enhance pregnancy rates following ART. Morphological evaluation is the most widely used method for embryo selection, constituting an excellent predictor of pregnancy success. However, clinical pregnancy rates remain around 35% (Wyns *et al.*, 2020), suggesting that there is still room for improvement. Time-lapse screening of embryo development (Armstrong *et al.*, 2019) or diverse molecular analyses performed on embryo biopsies or in the culture medium of the embryos (Mastenbroek *et al.*, 2011) have been proposed as complementary methods to assist embryo selection. For this purpose, cumulus cells (CCs) provide an interesting biological material on which to perform molecular analyses, as they are closely connected to the oocyte throughout folliculogenesis and oocyte maturation and are discarded prior to fertilization (Kordus and LaVoie, 2017).

Molecular analyses on CCs aiming to predict pregnancy success have been majorly based on transcriptomics analyses, leading to the identification of diverse putative markers for oocyte quality and subsequent embryo development (Iager *et al.*, 2013; Wathlet *et al.*, 2013; Borup *et al.*, 2016; Parks *et al.*, 2016; Artini *et al.*, 2017; Martínez-Moro *et al*., 2022). However, the different transcriptional markers identified vary greatly between studies, and others have failed to find a correlation between pregnancy success and gene expression in CCs, either for genes previously suggested as potential markers (Burnik Papler *et al.*, 2015) or in global transcriptomics analyses (Burnik Papler *et al.*, 2015; Green *et al.*, 2018). Compared to transcriptomics analyses, the evaluation of relative mitochondrial DNA (mtDNA) amount in CCs constitutes a faster, easier and less expensive molecular analysis, and it provides an easier-to-compare unique data value per sample, which makes it appealing for its implementation in routine IVF.

Several lines of evidence suggest that mtDNA content in CCs may be a good predictor for oocyte quality. During oocyte growth, mtDNA undergoes massive proliferation (Cao *et al.*, 2007; Cree, *et al.*, 2008) and oocyte mtDNA content has been consistently correlated to oocyte maturity during folliculogenesis and subsequently to fertilization outcome in animal models (El Shourbagy *et al.*, 2006; Lamas-Toranzo *et al.*, 2018). In humans, oocyte mtDNA content was found to be lower in cohorts of oocytes undergoing fertilization failure compared to cohorts displaying normal fertilization rates (Reynier *et al.*, 2001). Similarly, mtDNA content was reported to be higher in human zygotes compared to unfertilized oocytes (Santos *et al.*, 2006) and in *in vivo* matured human oocytes compared to *in vitro* matured oocytes (Zhao *et al.*, 2016), whereas a reduction in oocyte mtDNA has been associated with ovarian insufficiency (May-Panloup *et al.*, 2005) and aging (Chan *et al.*, 2005; Murakoshi *et al.*, 2013).

Given the essential roles of CCs during oocyte growth and maturation, a similar positive correlation between mtDNA content in CCs and oocyte quality may occur. Oocyte and CC mtDNA content have been positively correlated in human samples (Boucret *et al.*, 2015), and mtDNA content in human CCs has been reported to correlate positively with embryo quality, as assessed by morphology on Days 3 and 5 (Ogino *et al.*, 2016) or 2 (Desquiret-Dumas *et al.*, 2017). Furthermore, going beyond conventional embryo selection based on morphology, another article associated mtDNA content in human CCs with the odds of implantation (Taugourdeau *et al.*, 2019). In view of these findings, the objective of this study was to determine if mtDNA content in CCs is correlated to the developmental potential of its enclosed oocyte. To achieve this, we have analyzed mtDNA in human samples collected from IVF cycles showing diverse developmental competence, and in bovine oocytes, an animal model with some similarities to the human reproductive process.

## Materials and methods

### Collection of human samples

CCs were obtained at IVF Spain Madrid from IVF cycles between June 2018 and June 2020. The study was approved by the Ethical Committee from La Princesa University Hospital (Madrid) and all patients and donors were informed and agreed to participate in the study. The study was conducted on both autologous and heterologous (oocyte donation) embryo transfers meeting the following inclusion criteria: absence of uterine abnormalities; donor age ≤37 years; recipient age ≤50 years; and sperm count in ejaculate >2 million spermatozoa/ml. Only unfrozen oocytes and embryos not submitted to preimplantation genetic testing were included in the analysis. Characteristics of the patients included in the study are presented in Table I.

**Table I:** Patient characteristics in a study of mitochondrial DNA content of cumulus cells.

	Pregnant	Non-pregnant
Patient age (years)	36.77 ± 0.85	37.79 ± 0.78
Patient BMI (kg/m^2^)	24.37 ± 0.85	23.83 ± 0.88
Endometrial thickness (mm)	9.07 ± 0.35	9.35 ± 0.26
Donor age (years)	31.77 ± 1	32.97 ± 1.09
Donor BMI (kg/m^2^)	24.9 ± 0.9	23.34 ± 0.91

Data are shown as mean ± SEM, no significant differences were found between groups (Student’s *t*-test *P* > 0.05).

Cumulus–oocyte complexes (COCs) were obtained from both patients and donors stimulated by FSH treatment starting on the second menstruation day. Ovulation was induced by GnRH analog in the presence of three or more follicles larger than 17 mm (Melo *et al.*, 2009). Oocyte retrieval was performed 36 h after hCG injection by transvaginal ultrasound guidance. Prior to embryo transfer, endometrium was stimulated by the administration of 6 mg/day of estradiol (Meriestra^®^). Embryo transfer was performed only if the endometrium measured 7–13 mm (Prapas *et al.*, 1998). Luteal support started on the night of oocyte retrieval and involved the administration of 400 mg of progesterone every 12 h until a pregnancy test (Edwards *et al.*, 1984).

COCs were retrieved by follicular aspiration, washed and individually cultured in G-IVF Plus Media (Vitrolife, Göteborg, Sweden) (6.3% CO_2_, 5% O_2_, 89% N_2_ at 37°C). Denudation was performed individually in a hyaluronidase solution (Irvine) 2 h after COCs recovery. CCs detached from oocytes were collected from denudation medium, pelleted by centrifugation at 1500*g* for 10 min, snap frozen in liquid nitrogen and stored at −80°C until analysis. Oocytes were fertilized by ICSI in G-MOPS Plus medium (Vitrolife, Göteborg, Sweden). Spermatozoa were introduced in 7% polyvinylpyrrolidone solution to slow their movement. Following ICSI, the presumptive zygotes were cultured individually in Continuous Single Culture SAGE 1-Step™ medium (Origio Malov, Denmark) for 6 days, up to the blastocyst stage. Morphological score was used to select blastocysts for embryo transfer (Gardner *et al.*, 2015). Morphological score was similar for both groups transferred (P− and P+, see below). Pregnancy was assessed at the fourth or fifth week post-fertilization by fetal heart rate detection by ultrasound echography. Once embryo development was known, the previously stored CCs were allocated into three groups according to oocyte developmental potential: oocytes not developing to blastocyst (Bl−); oocytes developing to blastocyst but failing to establish pregnancy following embryo transfer (P−); or oocytes developing to blastocyst and able to establish pregnancy (P+).

### Collection of bovine samples

Bovine embryo production was performed following conventional protocols with minor modifications (Lamas-Toranzo *et al.*, 2020). Two independent experiments were conducted in bovine samples. The first experiment aimed to collect CC samples from oocytes exhibiting different developmental potential. Bovine ovaries were collected at slaughterhouse and transported at 35–37°C to the laboratory. COCs were obtained by aspiration of 2–8 mm follicles and selected using conventional morphological criteria (Hawk and Wall, 1994). COCs were matured individually in 40 µl drops of TCM-199 supplemented with 10% fetal calf serum and 10 ng/ml epidermal growth factor, and covered under mineral oil at 39°C and 5% CO_2_ in air with a humidified atmosphere for 24 h. Following maturation, CCs were removed individually by pipetting in medium supplemented with 0.1% hyaluronidase. CCs were collected from the media by centrifugation at 750 g for 5 min, snap frozen in liquid nitrogen and stored at −80°C until analysis. Denuded oocytes were individually inseminated with 10^6^ frozen-thawed bull spermatozoa/ml and incubated in 40 µl drops of Tyrode’s albumin lactate pyruvate medium (Parrish *et al.*, 1986) covered under mineral oil for ∼20 h in the same atmosphere conditions as above. Following IVF, presumptive zygotes were cultured individually in 10 µl drops of SOF (Synthetic Oviduct Fluid) medium (Holm *et al.*, 1999) at 39°C and in a 5% CO_2_ and 5% O_2_ humidity-saturated atmosphere. Cleavage and blastocyst rates were assessed at 48 h and 9 days post-insemination, respectively, in five independent replicates. Once the embryo development was known, the previously stored CCs were allocated to one of three groups according to oocyte developmental potential: oocytes not cleaving following IVF (Cl−); oocytes cleaving but not developing to blastocysts (Bl−); and oocytes developing to blastocyst (Bl+).

A second experiment aimed to determine if relative mtDNA amount in CCs correlated with that of the enclosed oocyte. CCs and oocytes samples were collected from *in vitro* matured bovine oocytes. CCs samples were collected as described above. For oocyte samples, the zona pellucida was removed by brief incubation in PBS pH 2 and zona-free oocytes were snap frozen individually and stored at −80°C until analysis.

### mtDNA analysis

CCs and oocyte samples were digested using a Picopure DNA Extraction kit (Applied Biosystems, San Francisco, CA, USA) following the manufacturer’s recommendation, using 20 µl and 10 µl of reagent per CCs and oocyte sample, respectively. In CCs, relative mtDNA quantification was determined as previously described (Lamas-Toranzo *et al.*, 2018). Relative quantification is required to compensate for the differences between samples in the number of CCs. Relative quantification was performed by using PCR primers that amplify a mitochondrial sequence (*MT-ND2* (mitochondrially encoded NADH dehydrogenase 2) and *COX1* (cytochrome c oxidase subunit 1) for human and bovine samples, respectively) and others to amplify a genomic sequence (*PPIA*, peptidylprolyl isomerase A) to adjust for the total amount of DNA present in the sample. Primer sequences are shown in Table II. Samples were run in duplicate, using 4 µl of digested sample in a 20 µl quantitative PCR (qPCR) reaction (Gotaq qPCR, Promega, Madison, WI, USA) in a MIC thermocycler (BioMolecular Systems, Upper Coomera, Australia). PCR conditions were optimized to achieve efficiencies close to 1 and then the comparative cycle threshold method was used to obtain relative values following 2^−ΔΔCq^ calculation, as described (Schmittgen and Livak, 2008). Absolute quantification was performed for oocytes following a similar method (Bermejo-Alvarez *et al.*, 2008), using the primers designed for mtDNA sequences and 3 µl of digested sample in a 20 µl PCR reaction. To compare groups, oocyte data were analyzed by the 2^−ΔCq^ method (Schmittgen and Livak, 2008).

**Table II:** Sequences for the PCR primers used to quantify mitochondrial DNA in human and bovine samples.

Gene	Species	Primer sequences (5′–3′)	Fragment size (bp)	GenBank accession no.
*MT-ND2*	Human	CAGCACCACGACCCTACTAC GGAGGGTGATGGTGGCTATG	194	NC_012920.1
*PPIA*	Human	TCTGCACTGCCAAGACTGAG CCCAGCTAAGGGCCAAGTTT	230	NC_0000007.14
*COX1*	Bovine	TTTGATGCTTGGGCCGGTAT GGAATGAGGGAGGGAGGAGT	265	NC_006853.1
*PPIA*	Bovine	CGGGATTTATGTGCCAGGGT CCAAAGTACCACGTGCTTGC	218	NC_037331.1

*MT-ND2*, mitochondrially encoded NADH dehydrogenase 2; *PPIA*, peptidylprolyl isomerase A; *COX1*, cytochrome c oxidase subunit 1.

The number of human samples analyzed was 109 (37, 38 and 34 for Bl−, P− and P+ groups, respectively). Experiments in the bovine model analyzed 120 samples in the first experiment (40/group) and 170 in the second (85 oocytes and their corresponding CCs). Statistical differences were analyzed by *t*-test or ANOVA using SigmaStat (Jandel Scientific, Leighton Buzzard, UK). *P*-values above 0.05 were not considered statistically significant.

## Results

**Figure 1.**
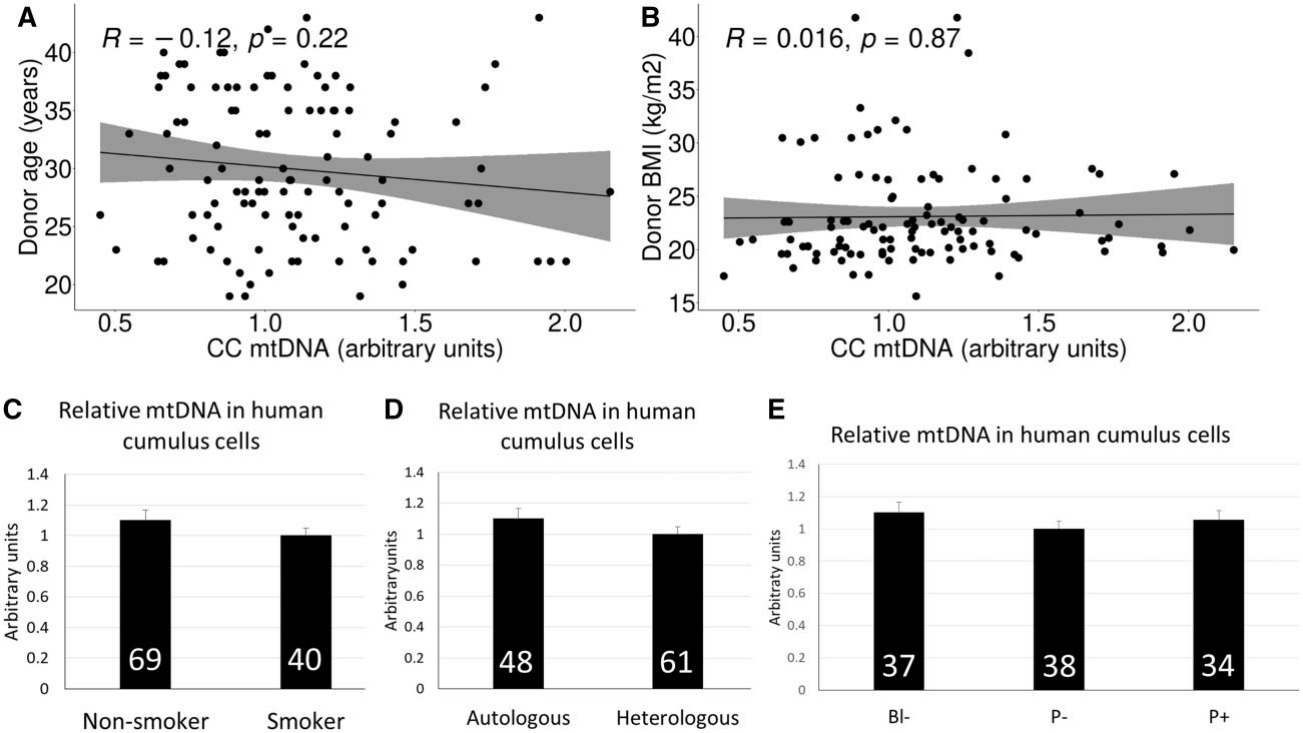
**Analyses of mitochondrial DNA (mtDNA) content in 109 samples of human cumulus cells (CCs).** (**A**) Donor age plotted against mtDNA content in human CCs. No correlation was found between both parameters. (**B**) Donor BMI plotted against mtDNA content in human CCs. No correlation was found between both parameters. (**C**) Relative mtDNA abundance in human CCs from donors who were non-smokers or smokers. No significant differences were found by Student’s *t*-test (*P* > 0.05). The number of samples analyzed per group is indicated within each column. (**D**) Relative mtDNA abundance in human CCs from autologous and heterologous (oocyte donation) cycles. No significant differences were found by *t*-test (*P* > 0.05). The number of samples analyzed per group is indicated within each column. (**E**) Relative mtDNA abundance in human CCs obtained from oocytes not developing to blastocysts (Bl−), developing to blastocyst but failing to establish pregnancy (P−) or establishing pregnancy (P+). Data are presented as mean ± SEM. No significant differences were found by ANOVA (*P* > 0.05). The number of samples analyzed per group is indicated within each column.

The analysis of relative mtDNA amount in 109 samples of human CCs revealed that this parameter was not associated with donor BMI or age (Fig. 1A and B). No differences in CCs mtDNA content were found between donors who were non-smokers or smokers (Fig. 1C) or between samples recovered from autologous or heterologous (oocyte donation) cycles (Fig. 1D). Recipient age, BMI and endometrial thickness were also similar between pregnant and non-pregnant groups (Table I). Thereafter, mtDNA content in human CCs was tested as a proxy for the developmental competence of their enclosed oocyte. mtDNA content in human CCs was remarkably similar between samples collected from oocytes not developing to blastocysts versus oocytes developing to blastocysts, irrespective of the pregnancy outcome (Fig. 1E), suggesting that this parameter provides a poor predictive value of oocyte developmental potential for *in vitro* embryo development. Similarly, relative mtDNA abundance did not vary between samples collected from oocytes ultimately establishing a pregnancy versus those developing to blastocyst but failing to establish pregnancy (Fig. 1D).

**Figure 2.**
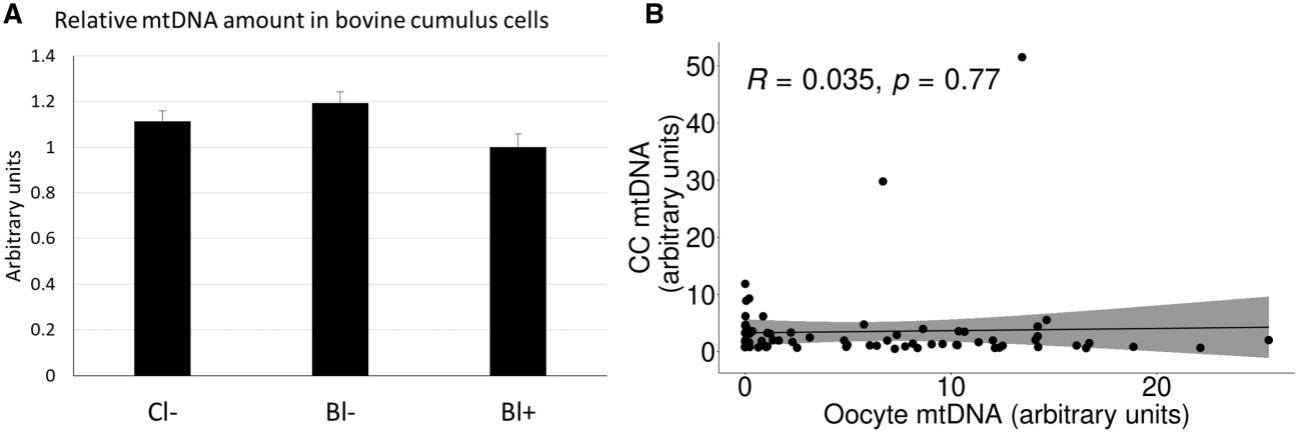
**Relative mitochondrial DNA (mtDNA) abundance in bovine cumulus cells does not vary according to oocyte developmental potential.** (**A**) Relative mtDNA abundance in bovine cumulus cells obtained from oocytes not cleaving (Cl−), cleaving but not developing to blastocyst (Bl−) or developing to blastocyst (Bl+). Data are presented as mean ± SEM. No significant differences among groups were found by ANOVA (*P* > 0.05). Forty samples were analyzed per group. (**B**) mtDNA content in bovine cumulus cells plotted against mtDNA content of their corresponding oocytes (85 samples). No correlation was found between both parameters.

Aiming to reduce the effects of possible confounders inherently associated with human samples, mtDNA analysis in CCs was also performed in the bovine model. Cattle share with humans a mono-ovulatory folliculogenesis and similar timing of embryo development, which together with the possibility of using ovaries collected from the slaughterhouse makes it an excellent model for experimentation in human IVF (Lamas-Toranzo *et al.*, 2018). As observed for human samples, relative mtDNA abundance in bovine CCs did not vary according to their developmental potential, being similar in non-cleaving oocytes, oocytes developing into cleaved embryos but arresting their development prior to blastocyst stage and oocytes developing into blastocysts (Fig. 2A). The use of an animal model also allows us to compare mtDNA content in oocytes and CCs, as mtDNA analysis in oocytes involves destroying the oocyte. mtDNA content in CCs was unrelated to that of their enclosed oocyte (Fig. 2B).

## Discussion

Previous analyses of oocyte mtDNA in cohorts of oocytes showing different developmental potential or in unfertilized oocytes versus zygotes have identified a positive correlation between mtDNA amount and oocyte quality in humans (Reynier *et al.*, 2001; Santos *et al.*, 2006; Zhao *et al.*, 2016) and experimental animal models (El Shourbagy *et al.*, 2006; Lamas-Toranzo *et al.*, 2018). Unfortunately, the analysis of mtDNA in oocytes is not compatible with subsequent development, while CCs are readily available following ICSI. In this sense, mtDNA analysis in CCs provides an alluring alternative method to select embryos based on their developmental potential. mtDNA analysis is less complex and faster than other molecular analyses, such as transcriptomics, yielding a result in a matter of hours, well before embryo transfer is performed, and thereby being readily applicable to IVF cycles without the need for embryo freezing. However, in contrast to previous observations (Ogino *et al.*, 2016; Desquiret-Dumas *et al.*, 2017; Taugourdeau *et al.*, 2019), we have not found a correlation between mtDNA content in CCs and the developmental potential of the enclosed oocyte in either human or bovine samples. The reason for such a discrepancy is unknown, but it may be caused by differences in mtDNA analysis or by the different criteria used to assess embryo developmental potential.

mtDNA content in human CCs has been associated with embryo developmental potential, inferred indirectly using morphokinetical parameters (Ogino *et al.*, 2016; Desquiret-Dumas *et al.*, 2017; Yang *et al.*, 2021). While this association is certainly interesting from a biological perspective, it does not provide any more information for embryo selection than conventional morphological selection. In other words, for a practical use, mtDNA analysis should provide additional predictive value of implantation rate to that already available by morphokinetical parameters. A previous study reported a positive correlation between mtDNA content in CCs and implantation potential (Taugourdeau *et al.*, 2019), but the dispersion of the reported data (215 ± 375 versus 59 ± 72 for implanting versus non-implanting, respectively) may make this possible correlation not readily applicable for embryo selection, as it would be difficult to establish a given threshold to predict implantation potential. As uterine receptivity plays a major role in implantation potential (Craciunas *et al.*, 2019), our experimental design aimed to identify a putative gradual increase in CCs mtDNA between oocytes not developing to blastocyst versus oocytes developing to blastocyst (unaffected by recipient parameters) and between oocytes developing to blastocyst failing to implant versus able to implant. Despite obtaining a small intra-group variability (<7% SEM), mtDNA content in human CCs was unrelated to oocyte developmental potential. In agreement with our data, two recent articles found no correlation between mtDNA in human CCs and morphological parameters on Day 3 or subsequent implantation potential (Liu *et al.*, 2021) or oocyte maturity, fertizability, embryo quality and pregnancy rates (Kumar *et al.*, 2021). To further test a possible relation between mtDNA amount in CCs and embryo development, experiments were conducted on the bovine model, where experimental conditions are tightly controlled, as no hormonal treatments are used prior to oocyte collection and all oocytes are fertilized by the same fertile male. Data obtained here from the bovine model also failed to link mtDNA content in CCs with subsequent embryo development and found no correlation between mtDNA amount in CCs and oocytes, further refuting the use of this parameter for embryo selection.

While CC mtDNA content was not found to be a good predictor of implantation potential and qPCR-based oocyte mtDNA analysis is not compatible with subsequent development, the analysis of mtDNA content in embryo biopsies may provide good predictive value. Elevated mtDNA in trophectoderm biopsies has been associated with poor blastocyst implantation potential (Diez-Juan *et al.*, 2015; Fragouli *et al.*, 2015), although others have failed to find such a correlation (Treff *et al.*, 2017; Victor *et al.*, 2017; Klimczak *et al.*, 2018; Scott *et al.*, 2020). The possible negative correlation between implantation potential and mtDNA content in embryo biopsies has been suggested to be caused by a compensatory mechanism for an insufficient or faulty mitochondrial pool at the end of oogenesis (May-Panloup *et al.*, 2016). However, embryonic mtDNA replication before implantation still remains a matter of controversy (Piko and Taylor, 1987) and a higher relative mtDNA in poor-quality embryos has also been suggested to be a consequence of degradation of genomic DNA (Ho *et al.*, 2018). In any case, the positive correlations reported for mtDNA in oocytes and different developmental parameters (Reynier *et al.*, 2001; Chan *et al.*, 2005; May-Panloup *et al.*, 2005; El Shourbagy *et al.*, 2006; Santos *et al.*, 2006; Murakoshi *et al.*, 2013; Zhao *et al.*, 2016; Lamas-Toranzo *et al.*, 2018) suggest that different strategies aimed at increasing or correcting the mitochondrial pool in the oocyte may help to improve ART outcomes (Craven *et al.*, 2017).

## Data availability

The data underlying this article will be shared on reasonable request to the corresponding author.
